# Analyzing Social Support from Facebook on Viral Suppression among Young Black Men Who Have Sex with Men Living with HIV: A Pilot Study

**DOI:** 10.3390/ijerph21101352

**Published:** 2024-10-12

**Authors:** Eleanor E. Friedman, Natascha Del Vecchio, Joseph A. Mason, Samantha A. Devlin, Jessica P. Ridgway, John A. Schneider

**Affiliations:** Chicago Center for HIV Elimination, University of Chicago, 5841 South Maryland Avenue, MC 5065, Chicago, IL 60637, USA

**Keywords:** HIV, young black MSM, Facebook, viral suppression

## Abstract

Social support has been associated with viral suppression among persons living with HIV (PWH). We examined cross-sectional data from young Black men who have sex with men to see if sociodemographic factors, medical history, and egocentric Facebook social support measures are related to viral suppression. Differences between participants were examined using Chi-square, Fisher’s exact, or Wilcoxon Rank Sum Tests, with significance set at *p* = 0.10. Degree centrality (*p* = 0.074) and Eigenvector centrality (*p* = 0.087) were significant, indicating that PWH with unsuppressed viral loads had more social connections. These results contrast prior studies in the literature. Further research on online social support for PWH is needed.

## 1. Introduction

Among people living with HIV (PWH), viral suppression promotes better health outcomes for individual patients (e.g., reductions in morbidity and mortality) and benefits the public (e.g., prevention of HIV transmission) [1]. Viral suppression is the final step in the HIV care continuum, a model that measures HIV awareness and care from diagnosis with HIV to adherence to treatment with antiretroviral therapy (ART) resulting in suppression of HIV RNA production [2]. In the United States, some demographic groups are more likely to be living with HIV, including African American/Black men who have sex with men (MSM) and young people (ages 13–34 years) [3,4]. In addition, African American/Black men living with HIV are less likely to be virally suppressed than other demographic groups [5].

Racial disparities in HIV diagnosis and viral suppression among PWH result from a complex interplay between individual-level vulnerabilities and systemic/structural barriers. Previous research has shown that multiple socio-environmental factors result in the difference in viral suppression between white MSM and Black MSM. These include lack of access to ART or inconsistent ART coverage, being unstably housed, having lower income, and marijuana use. Facilitators for viral suppression and better engagement in multiple steps in the HIV care continuum among MSM include social support and psychological coping; however, studies have demonstrated the complexity of social support for MSM living with HIV, especially among Black men [6,7].

Social support has proved beneficial for many diseases and health outcomes, including HIV. Previous research has indicated that social support is helpful for increasing engagement for multiple steps in the HIV care continuum, including reducing HIV stigma, increasing linkage to care, increasing retention in care, and viral suppression [8,9,10,11,12,13,14]. There are two broad ways in which social support is conceptualized to be beneficial to health. Structural social support, the number and characteristics of an individual’s social network (e.g., the breadth, density, and interconnectedness of one’s network), may protect against the negative effect of isolation. Alternatively, functional social support, the resources provided by a person’s social network (e.g., emotional support, socialization, financial assistance, and advice), can reduce life stress [15,16]. Of note, prior research has shown that Black and Hispanic MSM living with HIV have lower levels of functional social support than their white counterparts [7]. With the arrival of technology like cell phones, internet forums, dating websites, and social networking sites, new avenues for examining network structural support have emerged [17,18,19,20]. Prior research has indicated that young MSM living with HIV perceive and receive social benefits from online interactions on social media networking sites [21,22,23]. Additionally, previous work using egocentric network measures has examined anticipated advice and discussion around pre-exposure prophylaxis for HIV among Black women and has been integrated into a randomized controlled trial to increase HIV testing among MSM in China [24,25]. Furthermore, although social network interventions for HIV have been shown to improve engagement in HIV prevention and testing, few studies have previously examined if structural social support measured solely using a participant’s online social network actually influences HIV care continuum outcomes. We examined the effect of egocentric measures of social support derived from Facebook on PWH’s viral suppression. We analyzed data collected from young Black men who have sex with men (YBMSM) in the greater Chicago metropolitan area, examining how demographic factors, socioeconomic factors, medical history, and Facebook friend data are related to viral suppression.

## 2. Materials and Methods

Original project: The Neighborhoods and Networks (N2) Cohort Study sought to characterize the relationship among social and sexual network characteristics and risk for HIV infection, as well as HIV-related prevention and care behaviors, among YBMSM in Chicago, Illinois; Baton Rouge, Louisiana; and New Orleans, Louisiana. The methods of the N2 study have been described in detail in previous publications [26,27].

Current investigation: For this sub-analysis of the N2 study, we focused on participants who enrolled and provided informed consent in Chicago, examining data collected at the time of enrollment, from January of 2018 to February of 2019. We limited our analysis to YBMSM living with HIV who had both HIV viral load test results, as well as information collected from Facebook to characterize participants’ online social networks. Information taken from Facebook included a list of the participants’ friends that was used to create egocentric network measures, including the total number of friends, Eigenvector centrality, betweenness centrality, and degree centrality [28,29,30]. Centrality measures are used in social network analysis to assign a numeric score that represents the importance of people within their networks. As importance can be measured in multiple ways, different centrality measures have been created over time. Eigenvector centrality is a measure of the influence of a person within a network; betweenness centrality measures how often the participant is the shortest path between two friends within the network; and degree centrality measures the number of connections a person has to others in the network. If two egos provided the same name of a friend, we assumed that the friend was one held in common between these participants, rather than assuming that two different people with the same name had friendships among the study population.

Variables of interest: Demographic information collected at enrollment during the interview included race, ethnicity, and age. Additional social measures collected included lifetime incarceration history and lifetime history of exchanging sex for money or shelter. Finally, we examined clinical information including sexually transmitted infection (STI) history (syphilis, chlamydia, and gonorrhea positive test results < 90 days), as well as HIV screening test results and viral load measurements (within <90 days). We defined viral suppression as <200 HIV RNA copies/mL and unsuppressed as ≥200 HIV RNA copies/mL. Syphilis positivity was defined as a rapid plasma reagin test result of ≥1, which can indicate either past infection with syphilis or current infection with syphilis.

Statistical analysis: Differences between virally suppressed and unsuppressed participants were examined using Chi-square or Fisher’s exact tests or Wilcoxon Rank Sum Test for nonparametric tests. Due to the small sample size, significance was set a priori at 0.10 before conducting any analysis. Facebook data were cleaned using Python (including packages beautifulsoup4.9.3, soupsieve2.2.1, and pandas1.2.4). Analysis was completed using R Studio.

## 3. Results

We identified 59 YBMSM living with HIV who had both HIV viral load information and Facebook data. Most participants identified as non-Hispanic Black (81.36%), and the most common age group was 27 to 32 years old (44.07%). Few participants endorsed that they had ever been incarcerated (6.78%) or had exchanged sex for money or shelter (3.39%). Nearly half of all participants had a history of syphilis (45.76%), and around a fourth had a history of gonorrhea (22.03%). In terms of Facebook-derived measures, about half of the participants (50.85%) had more than 1500 Facebook friends (Table 1).

When examining the distribution of these variables between YBMSM who were virally suppressed and those who were unsuppressed, few variables showed differences between these groups (Table 1). At the significance level of ≤ to 0.1, we did not detect any differences for demographic factors, socioeconomic factors, STI history, or number of Facebook friends. We did see significant differences when examining the centrality measures of degree centrality (*p* = 0.074) and Eigenvector centrality (*p* = 0.087), indicating that PWH with unsuppressed viral loads had more connections to others than PWH with viral suppression and that PWH without viral suppression were more influential within the network than PWH with viral suppression. Additionally, differences between virally suppressed versus unsuppressed PWH regarding betweenness centrality approached significance (*p* = 0.123), once again indicating that PWH without viral suppression had shorter paths to other network members than PWH who were virally suppressed (Table 1).

## 4. Discussion

In this study, we found that degree centrality and Eigenvector centrality were higher in PWH with elevated HIV viral loads as compared to PWH who were virally suppressed. These results are largely in contrast to prior studies in the literature that suggest that greater social support is associated with adherence and viral suppression among PWH [7,31,32]. Importantly, we measured structural social support only using egocentric measures generated from a single social network site, as opposed to measuring social support using validated assessments for structural support, functional support, or both. Our choice of measurements may be responsible for the difference in our findings as compared to previous studies related to social support and antiretroviral therapy (ART) adherence or viral suppression. This is supported by Comfort et al., who examined egocentric social support measures among PWH and adherence to ART in Uganda and South Africa [33]. These authors measured social support using a range of egocentric measures, including total network size, number of friends, number of family members, and betweenness centrality. In Uganda, the only significant association was the number of same-gender network members, which was positively associated with ART adherence. In South Africa, significant associations included having more friends, which was negatively associated with ART adherence, as well as near significance (*p* = 0.1) for negative associations with betweenness centrality and also for the number of same-gender network members. The results from South Africa indicate that it is possible that in particular geographic contexts or among certain groups, measures of egocentric social support may not be positively aligned with HIV care outcomes.

It is also possible that our findings could have stemmed from a difference in support offered to PWH by online versus in-person connections. Studies have shown that unlike the support provided by face-to-face friendships, online social support—particularly that provided by friendships on Facebook—are not associated with certain health benefits [34]. For instance, Meshi and Ellithorpe found that increased social media use is significantly associated with decreased real-life social support and that only real-life social support is associated with reduced depression, anxiety, and social anxiety [35]. However, the findings of Comfort et al. were based on in-person social networks, and they also reported negative results for several egocentric network measures in South Africa, suggesting that the way social support is measured may be more important than if it is measured among in-person versus online networks. Additional studies could help elucidate this discrepancy.

Among YBMSM in the South, Sterrett-Hong et al. found “having close and supportive personal relationships” was helpful for each stage of the HIV care continuum [6]. These types of friendships may provide functional social support in a way that is less available in online relationships, for instance via in-person meet ups, physical contact, or through the direct provision of goods and services. Our findings may reflect the fact that our measures are based on the number and structure of friend groups rather than the perceived emotional closeness of these friendships. Unfortunately, we extracted friendship data from Facebook as a list of connections without additional contextual information (e.g., posts, other communications between specific connections/Facebook members) that is necessary to determine the exact nature of these friendships. It is also impossible to determine if these friendships are formed only among people online or if they cross the in-person and online spheres.

Regarding how MSM specifically use social media for support, there are several possible explanations for why network members with unsuppressed viral loads had more connections to others and were more influential within their social network. Much of the social support for PWH regarding HIV care continuum outcomes is predicated on the disclosure of HIV status [36]. It is possible that network members without viral suppression do not disclose their status to their online friends, and, consequently, the number of their online friends is unrelated to HIV care outcomes such as viral suppression. Recent work based on both online and offline social networks in China have demonstrated that MSM prefer to disclose their HIV status to online friends rather than their offline network of family, friends, and important people due to the “perceived threat” related to HIV stigma [17,37]. Previous research has shown that internalized HIV stigma is positively associated with HIV disclosure and negatively associated with ART adherence and viral suppression. It is possible that in our study we had participants who fell into two groups: those with internalized HIV stigma who had many superficial online friendships and elevated viral loads, and those who carefully maintained a few strong connections for the sake of overcoming potential stigma.

This study has several limitations, including a small sample size composed of a relatively homogenous population. This prevented us from conducting adjusted analyses of these data, limiting the robustness of our analysis. It is possible that the data we present here actually represent either sexual partnerships or romantic relationships as opposed to friend-based support. However, by using Facebook, as opposed to social media applications designed for dating or sex, we felt as though we were more likely to capture friendships that provide true social support. This analysis was limited by only examining online social network data from one platform, and therefore these findings related to social support cannot be extrapolated to other social media connections. Given the current social media landscape, multiple social media sites (i.e., Instagram, TikTok) and their networks may need to be included in future studies in order to provide a comprehensive understanding of the social support provided by online connections. Additional limitations include the fact that this was a cross-sectional study, meaning that viral suppression and Facebook friendships were measured at the same time, and the possible causality of these relationships is unclear. Lastly, it is possible that we overestimated the connectedness of the network with our assumption that the same name repeated among egos represented the same person.

## 5. Conclusions

This study examined the effect of egocentric measures of social support derived from Facebook on PWH’s viral suppression. We found that degree centrality and Eigenvector centrality were higher in PWH with elevated HIV viral loads as compared to PWH who were virally suppressed. Further studies that examine social support derived from online social network measures are needed to confirm these findings. If network members with elevated viral loads are confirmed to be more influential within their social networks, then social network strategies using online network opinion leaders (i.e., change agents with elevated viral loads) could be used to promote HIV engagement in care and medication adherence.

## Figures and Tables

**Table 1:** Characteristics of PWH with and without viral suppression.

Variable	Total (N = 59)	Virally Suppressed (N = 34)	Virally Unsuppressed (N = 25)	Chi-Square or Fisher’s Exact *p*-Value
Age (years) **				
21 to 26	10 (16.95%)	5 (14.71%)	5 (20.00%)	0.993
27 to 32	26 (44.07%)	16 (47.06%)	10 (40.00%)	
33 to 38	21 (35.59%)	12 (35.29%)	9 (36.00%)	
39 and up	1 (1.69%)	0 (0%)	1 (4.00%)	
Unknown	1 (1.69%)	1 (2.94%)	0 (0%)	
Race/Ethnicity				
Non-Hispanic Black	48 (81.36%)	26 (76.47%)	22 (88.00%)	0.0428 *
Hispanic Black	5 (8.47%)	5 (14.71%)	0 (0%)	
Non-Hispanic Black/Multiracial	2 (3.39%)	0 (0%)	2 (8.00%)	
No Response	4 (6.78%)	3 (8.82%)	1 (4.00%)	
Syphilis				
Positive	27 (45.76%)	15 (44.12%)	12 (48.00%)	0.933
Negative	14 (23.73%)	8 (23.53%)	6 (24.00%)	
Missing	18 (30.51%)	11 (32.35%)	7 (28.00%)	
Chlamydia				
Positive	9 (15.25%)	3 (8.82%)	6 (24.00%)	0.239
Negative	41 (69.49%)	26 (76.47%)	15 (60.00%)	
Missing	9 (15.25%)	5 (14.71%)	4 (16.00%)	
Gonorrhea				
Positive	13 (22.03%)	6 (17.65%)	7 (28.00%)	0.733
Negative	38 (64.41%)	23 (67.65%)	15 (60.00%)	
Missing	8 (13.56%)	5 (14.71%)	3 (12.00%)	
Ever Incarcerated				
Yes	4 (6.78%)	3 (8.82%)	1 (4.00%)	0.225
No	23 (38.98%)	10 (29.41%)	13 (52.00%)	
Missing	32 (54.24%)	21 (61.76%)	11 (44.00%)	
Exchanged Sex for Money or Shelter				
Yes	2 (3.39%)	1 (2.94%)	1 (4.00%)	0.281
No	34 (40.68%)	11 (32.35%)	13 (52.00%)	
Missing	33 (55.93%)	22 (64.71%)	11 (44.00%)	
Number of Facebook Friends				
Under 500	14 (23.73%)	9 (26.47%)	5 (20.00%)	0.839
500 to 999	10 (16.95%)	5 (14.71%)	5 (20.00%)	
1000 to 1499	4 (6.78%)	3 (8.82%)	1 (4.00%)	
1500 or More	30 (50.85%)	16 (47.06%)	14 (56.00%)	
Missing	1 (1.69%)	1 (2.94%)	0 (0%)	
Facebook Degree Centrality	0.098 (0.056–0.224)	0.084 (0.056–0.163)	0.203 (0.063–0.294)	0.074 *
Facebook Eigenvector Centrality	0.048 (0.019–0.110)	0.041 (0.016–0.074)	0.097 (0.024–0.142)	0.087 *
Facebook Betweenness Centrality	0.0036 (0.0004–0.0168)	0.0025 (0.0001–0.0083)	0.0090 (0.0009–0.0193)	0.123

* Fisher’s exact test was used. ** In calculating the Chi-square value, groups of ages 21 to 32 and 33 and up were used. Additionally, one participant of an unknown age was removed.

## Data Availability

As these data contain sensitive information, a fully de-identified version of the dataset is available on request from the authors.
